# The role of cognitive control affects memory encoding: evidence from subsequent memory paradigm

**DOI:** 10.3389/fpsyg.2025.1669174

**Published:** 2026-01-22

**Authors:** Yun Chen, Gaohui Jin, Shuai Yang

**Affiliations:** 1 College of Education Science, Hubei Normal University, Huangshi, China; 2 School of Electrical Engineering and Automation, Hubei Normal University, Huangshi, China; 3 School of Journalism and Communication, Hubei University of Economics, Wuhan, China

**Keywords:** cognitive control, inhibition, memory encoding, subsequent memory effect, task switching

## Abstract

Memory and cognitive control are fundamentally intertwined, but their interactions have only recently attracted researcher attention and the underlying mechanism of the interactions remains to be further explored. Several theoretical explanations for the influence of cognitive control on memory encoding include common-resource account, interactive model, and stage-specific encoding account. Specifically, common-resource account advocates that cognitive control sub-components compete for limited cognitive resources during memory encoding. Interactive model highlights the importance of selective attention and stage-specific encoding account emphasizes specific processing stage where cognitive control plays a role in memory encoding. By combing the existing literature, we found that different cognitive control sub-components exert differential effects on memory encoding. In other words, cognitive control can either enhance or impair memory encoding depending on the specific task demands and context. Firstly, compared to trials in which the current task is the same as the preceding task (repetition trials), the trials where current task is different from the preceding task (switch trials) will impair the memory encoding of target stimuli and enhance the memory encoding of distractor. Secondly, inhibition can facilitate memory encoding solely when the interference detecting from competing stimuli (i.e., Stroop task) rather than from stimulus and response (i.e., Simon task). Thirdly, both proactive and reactive inhibition possess the possibility to impair memory encoding. Future research could delve into this phenomenon from the perspective of exploring how multiple cognitive control integration influences memory encoding.

## 1 Introduction

In our daily life, we experience many events which we subsequently remember or forget. In the field of memory, the *subsequent memory paradigm* is often used to explore memory encoding and retrieval (Jia et al., 2021; Wang and Egner, 2022; Wiemer et al., 2021; Shi et al., 2020), in which participants are required to remember a series of items and then followed by a memory test. According to the results of the test, if items were correctly recognized, they were defined as remembered items. Otherwise, they were determined as forgotten items. In recent years, researchers have found that the recruitment of cognitive control not only affects the performance of current task but also the memory encoding process reflected by the subsequent memory recognition performance. In these studies, participants were asked to switch between two tasks or inhibit response or other irrelevant stimuli, in which trial-unique items were presented. Subsequently, a recognition memory tests for items presented in repeat/switching trials or congruent/incongruent trials (Chiu et al., 2021; Richter and Yeung, 2012). Behavioral studies have demonstrated that different sub-components of cognitive control have a positive or negative influence on memory encoding (Chiu et al., 2021; Chiu and Egner, 2015b; Muhmenthaler and Meier, 2021a; Reynolds et al., 2004).

Cognitive control refers to the ability to adopt goal-directed behaviors, including the processes that inhibit interference from goal-irrelevant information, detect and resolve conflict, and update behaviors for adapting to complex and variable environments (Botvinick et al., 2001; Diamond, 2013; Monsell, 2003). Existing studies generally perceived that cognitive control comprises three core components—cognitive flexibility, working memory, and inhibition— that can be measured by different paradigms, such as, task switching paradigm, Go/No Go task, and Stroop/Flanker task. Especially, the task-switching paradigm is effective because it allows for the manipulation of working memory and stimulus, and response level interference and response inhibition demands within the same paradigm (e.g., Meiran et al., 2000).

Given this, we review and summarize the relevant theories and findings from subsequent memory paradigm to provide beneficial references for future researchers (shown in Table 1). To ensure a comprehensive scientific summary, the following criteria were employed. The key words used for the literature search were “cognitive control,” “executive function,” “task switching,” “inhibition,” “interference,” “memory,” “memory encoding” and “subsequent memory.” Articles were sourced from three databases: Web of science, PubMed, and Science Direct with the search period spanning from 2000 to 2025.

**Table 1:** The main findings in previous relevant studies.

**Author/year**	**Type**	**Paradigm**	**Stimuli**	**Task**	**Memory measure**	**Main findings**
Reynolds et al. (2004)	fMRI	Task switching	Word	MAN-NAT classifications (human-made vs. natural) LRG-SMA classifications (larger vs. smaller than a standard computer monitor)	Old/New recognition test	Significantly more words were recognized from the single-task condition than from the tasks-witching condition
						LIPC showed a standard increase in activity associated with subsequently remembered words relative to subsequently forgotten words
						LIPC activation was greater in the task-switching relative to the single-task condition, although items encountered in the task-switching condition were not remembered as well as those encountered in the single-task condition
Richter and Yeung (2012)	Behavioral	Cue Task switching	Picture/word	Object classifications (human-made vs. natural) word classifications (abstract vs. concrete)	Memory ratings (6-point scale)	Memory ratings were higher for attended items
						Memory ratings for attended items were worse on switch trials than on repeat trials
						Memory ratings for unattended items were better on switch trials than on repeat trials
						RTs and error rates increased as memory selectivity decreased on switch trials
Richter and Yeung (2015)	Behavioral	Cue Task switching	Picture/word	Object classifications (human-made vs. natural) word classifications (abstract vs. concrete)	Memory ratings (6-point scale)	The difference in memory ratings for attended and unattended items (i.e., memory selectivity) was reduced on switch trials
						The memory ratings for repeat trial items increased when CSI was 1,000 ms compared to when CSI was 5 ms
						Memory ratings for attended items were increase in the 5 ms CSI condition than the 1,000 ms CSI condition
Richter and Yeung (2016)	ERP	Cue Task switching	Picture/word	Object classifications (human-made vs. natural) word classifications (abstract vs. concrete)	Memory ratings (6-point scale)	Higher memory ratings for attended than unattended items; RTs increased with decreasing memory selectivity
						After cue onset 600-900 ms, high memory selectivity trials were characterized by more negative voltages over posterior than trials with low memory selectivity
						After stimulus onset 900-1200 ms, high memory selectivity elicited more positive waves than low memory selectivity
Muhmenthaler and Meier (2019)	Behavioral	Alternating Task switching	Picture or word	EXP1: picture animals classification (mammals vs. birds) word objects Classification (musical instruments or kitchen utensils) EXP2: picture classification (smaller or bigger than a soccer ball vs. living or non-living)	Old/new judgment and remember/know judgment	Memory was significant better for repeat than for switch trials when the stimuli were univalent (EXP1) and bivalent (EXP2)
						More *remember* responses were associated with repeat than with switch trials (both EXP1 and EXP2)
						The effect of better memory for repeat than for switch trials was much stronger with bivalent stimuli (EXP2) than with univalent stimuli (EXP1)
Muhmenthaler and Meier (2021a)	Behavioral	Alternating Task switching	Word	Animals classification (mammals vs. birds) and objects classification (musical instruments or kitchen utensils)	Free recall test	Memory for target was significant better for repeat than for switch trials in all experiments
						Memory for distractor on switch trials was significantly better than for repeat trials, while this effect disappeared when there involved response-category conflict and stimulus-category conflict
						Enhanced memory performance for incongruent compared to congruent targets only when the conflict stimuli were presented in pure response-category conflict blocks
Muhmenthaler and Meier (2021b)	Behavioral	Flanker task	Picture	Animals classification (mammals vs. birds) and objects classification (musical instruments or kitchen utensils)	Old/new judgment and remember/know judgmment	Better memory performance for incongruent compared to congruent targets
						No congruence sequence effects for target
Muhmenthaler and Meier (2024)	Behavioral	Flanker task	Picture	Animals classification (mammals vs. birds) and objects classification (musical instruments or kitchen utensils)	Old/new judgment and remember/know judgmment	More incongruent targets were remembered than congruent targets in pure block (all the trials of a task block were either congruent or incongruent) the results showed better memory for congruent than incongruent flankers in pure blocks
						The results showed better memory for incongruent than for congruent in mixed block (congruent and incongruent trials were presented intermixed)
Chiu and Egner (2016)	Behavioral	Cue Task switching	Picture/word	Categorizing words (abstract vs. concrete) categorizing objects in the images (living vs. nonliving)	Memory ratings (4-point scale)	Better memory for distractors on switch trials than repeat trails in the intermittently irrelevant distractor condition
						The memory advantage for distractors on switch trials disappearing in the always-irrelevant distractor condition
Padovani et al. (2013)	ERP	Cue-task switching	Word	Word was animate or inanimate vs. word was neutral or emotional	Sure old/unsure old/sure new/unsure new	No significant memory difference between repeat and switch trials
						Prestimulus −2~-1s, remember elicited more frontal negative than forgotten only in repeat trials
						Prestimulus −1~0s, remember elicited more frontal negative than forgotten only in switch trials
Chiu and Egner (2015a)	Behavioral	Go/nogo task or stop signal task or Yes/No task	Male and female face stimuli	Response for only male or female (eg., go for male and nogo for female)	“Definitely new,” “probably new,” “probably old,” or “definitely old”	Memory was impaired for stimuli presented during no-go and stop trials compared with those presented during go trials
						*inhibition-induced forgetting* was not attributable to event congruency
Chiu and Egner (2015b)	fMRI	Go/nogo task	Male and female face stimuli	Response for only male or female (eg., go for male and nogo for female)	“Definitely new,” “probably new,” “probably old,” or “definitely old”	Memory wasworse for no-go than for go stimuli
						Brain activity during encoding of subsequently forgotten no-go trials was lower than subsequently remembered no-go trials
Chiu et al. (2021)	Behavioral	Picture-word interference task and go/no go task or stop signal task	Male and female faces/word	Judgement the face was male or female while ignoring the word, and response for only male or female	Memory ratings (4-point)	Memory for no-go trials was lower than memory for go trials
						Memory for incongruent trials was higher than memory for congruent trials
						Memory for stop trials was significantly lower than memory for go trials
						No interaction between response and congruency
Krebs et al. (2015)	fMRI	Face-word Stroop task	Male and female faces/word	Judgement the face was male or female while ignoring the word	Memory ratings (4-point)	incongruent trials yielded significantly better memory tares compared with those from congruent and neutral trials.
						Incongruent compared with congruent and neutral trials elicited several robust activation foci in the medial and lateral frontal cortex, including bilateral presupplementary motor area (pre-SMA) and dACC, bilateral dlPFC, and right inferior frontal gyrus
Ptok et al. (2019)	Behavioral	Backward compatibility effect (BCE) response priming and the psychological refractory period (PRP) paradigm	word	Task 1: categorizing word stimuli as bigger or smaller than the computer monitor Task 2: categorizing shapes into star or diamond vs. circle or pentagon	Old/new judgment	Memory benefit can be produced by semantic incongruency priming (EXP4 to EXP6)
						Lacking memory benefit when we prime response representations
Lee and Cho (2019)	Behavioral	Go/no-go task	Angry/neutral Male and female faces	Response for only male or female (eg. go for male and nogo for female)	Old/new judgment	no significant difference between go and no go trials when involved angry face
						D-prime for faces that were presented as go trials was significantly higher than that for that presented as no-go trials when the angry or all neutral faces were presented within a block
						That were presented as no-go cues inhibition-induced forgetting was negated only in the group in whom responses to angry faces were inhibited when participants needed to respond to the emotional expression of a face not gender
Jimenez et al. (2020)	Behavioral	Face-word Stroop task	Male and female faces/word	Judgement the face was male or female while ignoring the word	Sure not/believe not/believe yes/sure yes	No significant difference between congruent and incongruent trials
						A higher rate of high-confident recognition responses to faces presented under incongruent conditions selectively when they followed another incongruent trial, but not when they occurred after a congruent one
Rosner et al. (2014)	Behavioral	Naming task	word	Read aloud for one color stimuli	Old/new judgement and remember/know judgment	Recognition memory was better for incongruent than for congruent items (EXP1), even the there was no-distractor at test condition (EXP2)
						Recognition memory was better for the interleaved than for the separated items, which were two different types of “incongruent” items
Liu et al., 2016	ERP	Simon task	Chinese Chengyu words	Judgement the word was positive or negative	Old/new judgement	There was no difference in d' indexes between words encoded in congruent and incongruent trials
						The retrieval of positive words was more accurate than negative words encoded in the congruent condition, but not in the incongruent condition
						The memory word encoded in the incongruent trials elicited less negative EPN/FN and greater LPC amplitude than those encoded in the congruent trials

Attended items = task-relevant target items; unattended items = task-relevant distractors.

## 2 Theoretical accounts

### 2.1 Common-resource account

The common-resource account, also known as the resource-limitation account or the resource competitive account, proposes that the control process and the memory process compete over shared, capacity-limited resources (Craik et al., 1996; Otten and Rugg, 2001). If performing a task requires cognitive control, there are fewer resources available to encode information into long-term memory, manifested as impaired memory. This view is supported by several studies (Chiu and Egner, 2015b; Otten and Rugg, 2001). In the dual-task paradigm, researchers found that the execution of a more challenging secondary task exerts a greater detrimental impact on the encoding of stimuli in the primary task compared to the execution of a simpler secondary task (Uncapher and Rugg, 2005; Kensinger et al., 2003). Otten and Rugg (2002) argued that the processes involved in encoding and those managing control task sets compete for resources, leading to reduced allocation for encoding and thus poorer memory performance in trials with high control demands. Within the task-switching paradigm, task switching, in contrast to task repetition, requires additional cognitive resources to reconfiguration task sets (Rogers and Monsell, 1995) or inhibition the task set from the preceding trial (Allport et al., 1994). Consequently, during task switching, the requirement for cognitive control increases, thereby limiting the resources available for encoding target stimuli and leading to reduced memory performance. In the context of Go/No Go task, Chiu and Egner (2015b) also observed a significant decline in stimulus detection capability when reactive inhibition was necessitated, as the perceptual encoding resources were diverted to the No-Go trials to facilitate reactive inhibition.

### 2.2 Interactive model

The interactive model of control and memory, proposed by Richter and Yeung (2015), challenges the common-resource account by suggesting that the engagement of specific types of cognitive control can redirect attention either toward or away from item encoding, thereby enhancing or impairing memory for the item. In their earlier study, Richter and Yeung (2012) observed that memory selectivity—defined as the difference in memory ratings between target and distractor stimuli—was diminished in switch trials compared to repeat trials, indicating a negative correlation between control demands and memory selectivity. Furthermore, they demonstrated that an increase in top-down control also enhanced memory selectivity (Richter and Yeung, 2015). In their experiment, top-down control was manipulated via the cue-stimulus interval (CSI). The findings revealed that participants exhibited not only improved performance but also greater memory selectivity in the long CSI block (1,000 ms) compared to the short CSI block (5 ms). This improvement was attributed to the more effective allocation of attention to the target stimulus rather than the distractor when top-down control was heightened in the long CSI condition. Therefore, the interactive model is posited that during task switching, the increased demands on cognitive control influence memory encoding by diminishing attention allocation to task-related information and dispersing attention toward task-irrelevant information. This results in a reduction of selective processing of task-related stimuli, rather than a general decline in memory for all information. Contrary to the common-resource account, the interactive model emphasizes the importance of selective processing in cognitive control and memory (Richter and Yeung, 2012, 2015).

Further research indicates that participants' control abilities can influence memory encoding. Using the same task-switching paradigm as Richter and Yeung (2012), Brito et al. (2016) observed no difference in memory for target stimuli between simultaneous bilinguals and monolinguals; however, simultaneous bilinguals exhibited significantly poorer memory for non-target stimuli. These findings indicate that individual differences in attention control do affect memory for both target stimuli and non-target stimuli. Additionally, they found that the number of errors in switching trials was negatively correlated with the subsequent memory for target stimuli and positively correlated with the memory for non-target stimuli. That is to say, participants with stronger attention control (lower error rate) can remember more target stimuli and fewer non-target stimuli.

In contrast, Dubravac and Meier (2021) employed transcranial direct current stimulation (tDCS) over the parietal cortex during study phases where participants alternated between picture and word judgment tasks. They tried to establish a causal link between the activity of the dorsal attention network (P3) and the ventral attentional network (P6), which supports top-down and bottom-up attentional orienting and memory encoding. While the result showed that the different polarity of stimulation did not influence performance during task switching, nor did they affect subsequent memory performance. It could have meant the role of attention control in cognitive regulation and memory encoding may extend beyond the parietal cortex to also encompass the functions of the prefrontal lobe.

Furthermore, according to the conflict control model, when task-irrelevant stimuli cause a conflict representation, an up-regulation of top-down attention will be initiated to process target information more effectively, thereby mitigating the interference caused by task-irrelevant information (Botvinick et al., 2001). Therefore, the enhancement of memory for target stimuli presented in incongruent trials in the Stroop task is attributed to conflict resolution, which heightens attention to task-related stimuli rather than diverting cognitive resources from perceptual processing. This finding was corroborated by Rosner et al. (2018). In their experiment, participants were presented with green and red words and required to read the red words aloud while ignoring the green words. If the green and red words were identical, it constituted a repeat condition; if they differed, it was not repeated condition. The results indicated that response times were faster when the word was repeated compared to when it was not repeated.

### 2.3 Stage-specific encoding account

Unlike interactive model, stage-specific encoding account proposed that enhanced memory is not universally observed across all conditions of selective attention enhancement. In Ptok et al. (2019) studies, they conducted six experiments in which conflict was introduced at different stages, such as sensory/perceptual, categorization, response selection, and motor. The results indicated that recognition memory performance improved when semantic conflict arose between the distractor and target stimuli compared to congruent stimuli, suggesting that conflict during the categorization stage enhances memory. Conversely, memory facilitation was absent for incongruent stimuli when distractors induced response conflict, indicating that conflict during the response selection stage does not enhance memory. These findings were replicated and demonstrated by pupil dilation measures. They observed that slower reaction times and larger pupil dilation in incongruent trials with both semantic and response distractors compared to congruent trials, while memory improved in stimuli with semantic conflict. Additionally, when subjects were encouraged to focus on more information across all trials (with active control applied to all trials rather than just inconsistent trials), differences in pupil diameter and subsequent memory performance between high and low semantic conflict were reduced (Ptok et al., 2021).

This account is supported by Chiu et al. (2021). They conducted a study where participants judged the gender of the face pictures paired with “male” or “female” words. So, the semantic conflict was created when the face and word genders were incongruent. Participants were instructed to press a button for one gender and not respond to the other (Experiment 1) or to refrain from responding when a stop signal appeared (Experiment 2). The results demonstrated a clear congruency effect (more error rate and response time on incongruent trials than on congruent trials) and response inhibition effect during the study phases. However, the requirements for response inhibition and interference inhibition exerted opposing influences on subsequent memory. Specifically, memory was enhanced for stimuli exhibiting semantic conflict between targets and distractors, while it was impaired for stimuli necessitating response withholding or cancellation. Notably, there was no interaction between response suppression and conflict control on subsequent memory. This indicates that the two types of cognitive control, both related to attentional control, independently affect subsequent memory. This furtherly illustrates that the cognitive system may achieve its objectives through sequential processing stages.

In summary, the common-resource account highlights the limitations of cognitive resources, suggesting that competition is inevitable when faced with diverse cognitive demands. Conversely, the interaction model emphasizes the significance of selective attention in determining whether a stimulus is encoded into long-term memory. The stage-specific encoding theory advances the understanding of information processing by positing that cognitive control exerted at particular processing stages, such as the categorization stage, is crucial for influencing memory encoding. Furthermore, it suggests that various forms of cognitive control are executed across different processing stages.

## 3 Behavioural findings on cognitive control and memory encoding

### 3.1 Task switching and memory encoding

Task switching as a component of cognitive control, the switching cost is regarded as an indicator of flexibility, which is measured by the differences between trials where the task remains the same (repeat trials) and those where the task changes (switch trials) (Allport et al., 1994; Yeung et al., 2006). Existing researchers have found that task switching can impair memory. One typical result found that significantly more words were recognized from the sing-task than from the task-switching block (Reynolds et al., 2004). It meant, compared with the single-task block, the task-switching block demanded more attentional resources to update task sets (Rogers and Monsell, 1995) resulting less resources to encode the items. Furtherly, Richter and Yeung (2012) found that switching impaired subsequent memory was selective. In their experiment, participants switched between picture (classifying the objects as natural or human-made) or word task (classifying the words as abstract or concrete), with one word and picture presented together across trials resulting that the items could be divided into target stimuli (task-relevant) and distractors (task-irrelevant) according to the cues. In subsequent memory recognition, the memory performance for target stimuli was worse in switch trials as compared with repeat trials, whereas the memory for distractors was better in switch trials than in repeat trials. Later studies corroborated these findings (Chiu and Egner, 2016; Richter and Yeung, 2015), even when a free recall test was adopted instead of a recognition test (Muhmenthaler and Meier, 2021a, experiment 1). In addition, recent studies have shown that task switching impaired memory encoding target stimuli even for univalent stimuli, and this effect was increased when the material involves bivalent stimuli (Muhmenthaler and Meier, 2019).

According to the above findings, Richter and Yeung (2012) highlighted selective processing may be a key factor. Specially, task switching impaired selectivity reflected from the increased demands for cognitive control. To examine the factor, they manipulated cue-stimuli interval (CSI) (Richter and Yeung, 2015). As expected, memory ratings did not differ across switch and repeat trials when the CSI was short (5 ms) whereas the memory ratings for target stimuli improved for switch and repeat trials when the CSI was increased as 1,000 ms. Furthermore, Chiu and Egner (2016) found that the memory was better for switch trials than for repeat trials was probably due to task inertia, since the task-set of the previous trial sustained activation, resulting in the better memory for distractors.

Lately, the researchers conducted a comparative analysis of memory performance under proactive and reactive control conditions (Shi et al., 2020). In the proactive control condition, participants were able to prepare the task set before switching, as the cue was presented before the target. Conversely, in the reactive control condition, the cue and target were presented simultaneously. The study revealed that items encoded under the proactive control condition exhibited superior recognition performance compared to those under the reactive control condition. Furthermore, the influence of physical activity on the differences in subsequent memory between the two conditions was mediated by variations in individual cognitive control capabilities.

In summary, compared to task repetition, task switching can impair memory for target stimulus, but promote the memory for distractors.

### 3.2 Inhibition and memory encoding

Inhibitory control, or inhibition, refers to the ability to suppress irrelevant information, impede dominant response or inappropriate behavior in accordance with current goals (Brydges et al., 2012; Diamond, 2013). It is generally acknowledged, inhibition is classified as interference inhibition and response inhibition based on whether response was necessary. Interference inhibition, also known as attention inhibition or conflict inhibition, pertains to the inhibition of interference arising from competing stimuli, as observed in tasks like the *Stroop* and *Flanker* paradigms; Whereas, *response inhibition* involves the suppression of a pre-potent response, as exemplified by the interference observed in tasks like the *Go/No-Go* and *Stop Signal* paradigms. Depending on the specific characteristics of the task, response inhibition can be separated into reactive inhibition and proactive inhibition (Brydges et al., 2012; Cunillera et al., 2014). Proactive inhibition might include prior knowledge regarding the probability of stopping or which response should be stopped if a stop signal is presented (Zandbelt et al., 2010; Aron and Verbruggen, 2008). For example, the Go/No-Go task necessitated participants to promptly react to target stimuli while refraining from responding to non-target stimuli, in which the process involves internally driven. In contrast, reactive response inhibition takes place after recognizing the need to stop (Aron, 2011; Braver, 2012). For example, in the Stop Signal task, participants respond to “Go” stimuli, but they are required to stop responding if the stimulus is succeeded by a “Stop” signal. In this particular scenario, the response inhibition is driven by an external signal.

#### 3.2.1 Interference control and memory encoding

In studies related to interference or conflict inhibition and memory encoding, researchers have mainly used the Stroop task. Krebs et al. (2015) used the face-word Stroop task, in which male or female face pictures were presented together with the words “women” or “men” or “house” to form a congruent, incongruent, or neutral condition. During the memory encoding phase, incongruent trials had longer response time and error rates than congruent trials, but following face picture recognition, better memory performance occurred in incongruent trials in contrast to congruent and neutral trials. Latterly, Davis et al. (2019) manipulated the proportions of congruent and incongruent trials and found that the memory for stimulus presented in incongruent trials was better than in congruent trials and was robust and insensitive to list context.

Furthermore, according to *conflict monitoring theory*, conflict signals induced by incongruent trials trigger a temporal up-regulation of cognitive control that immediately improves its attention to the target stimulus upon encountering a conflicting trial, and this increase of cognitive control does not decline completely after a single trial but tends to have a gradual and cumulative effect (Botvinick et al., 2001). Jimenez et al. (2020) also used the face-word Stroop task to investigate whether memory enhancement was expressed in the trial (N+1) after the conflict trial (N). The results did not find an enhancement in behavioral performance as well as memory performance after incongruent trials compared with congruent trials. That is, the enhanced cognitive control triggered by conflict does not render long-lasting effects to promote memory encoding. In addition, Rosner et al. (2014), (2015) used a naming task, in which two words presented spatially interleaved in either red or green color, and participants were asked to read aloud the word printed in one of two colors ignoring another colored word. The recognition results of both studies showed that subsequent memory for target words was better when the target was presented with an incongruent distracter contrast to those identical. From above-mentioned studies, it is clear that in both the face-word Stroop and the color-word Stroop tasks, the memory encoding for the target stimulus was enhanced when there was interference or conflict with the target stimulus.

Recently, the findings have confirmed that inference can enhance memory encoding, as demonstrated in the Flanker task (Muhmenthaler and Meier, 2021b, 2024). In standard Flanker task, individuals respond to the central element within a sequence of stimuli (Eriksen and Eriksen, 1974). Similarly, the researchers used pictures as stimuli (Muhmenthaler and Meier, 2021b). When the target and flanker stimuli belonged to the same category (e.g., different birds), the condition was deemed congruent. Conversely, it was considered incongruent when the target and flankers were from different categories (e.g., a bird flanked by mammals). The subsequent memory test showed better memory performance for incongruent compared to congruent targets. Furtherly, they investigated the memory performance concerning task-irrelevant distractors. Consistent with Muhmenthaler and Meier (2021b), the identical targets and flankers were used, with congruent and incongruent trials presented in pure blocks and mixed blocks. The subsequent memory performance revealed that more targets were remembered in incongruent trials compared to congruent trials within the pure block, but not in the mixed block. Furthermore, in the mixed block, more incongruent flankers were remembered compared to congruent flankers (Muhmenthaler and Meier, 2024).

However, not all interference or conflict can enhance memory encoding. Ortiz-Tudela et al. (2018) used a spatial cueing paradigm in which participants generated a spatial expectation about the target location depending on the cue. Although reaction times were slower for words presented in invalidly cued location in contrast to those in validly cued locations, there was no evidence that a mismatch of spatial expectations could improve memory encoding. Similarly, Liu et al. (2016) used a modified Simon paradigm and participants were required to make a positive or negative judgment with their left or right hand respectively, on Chinese Chengyu words which would appear on the left or right side of the screen. At the encoding phase, incongruent trials showed worse performance relative to congruent trials. But subsequent memory did not perform better than those presented incongruent trials than congruent trials.

Consequently, the impact of interference or conflict control on memory encoding is inconsistent. The divergence is probably due to the difference between conflict types. The conflict in the Stroop task is stimulus-stimulus interference, while the conflict in the Simon task belongs to stimulus-response interference (Martínez-Pérez et al., 2020; Wang et al., 2014), in which different neural were activated leading to above inconsistent results.

#### 3.2.2 Response inhibition and memory encoding

The Go/No-Go and Stop-Signal Task have been mainly used to measure the response inhibition ability. Chiu and Egner (2015a) used the images of faces (e.g., male and female) as items. In the experiments, participants were asked to make Go response to images of one gender and the No-Go response to another gender in the Go/No-Go task, and to press the “V” key for images of one gender and press the “N” key for another gender while must to stop responding when the stop signal sound appeared in the Stop-Signal Task. The following surprise recognition memory results showed in both tasks, recognition performances were worse for stimuli presented during no-go and stop trials compared with those presented during go trials. Lee and Cho (2019) asked participants to perform a go or nogo response for images of one of two gender faces with emotionally charged (e.g., angry or neutral). Memory was impaired for items presented in the nogo trials in contrast to go trials when the emotional faces were presented in different blocks. However, when participants had to either press the button for angry faces without pressing button on neutral faces or reverse, the memory for nogo items was impaired only when they need to respond to angry faces not to neutral faces.

In conclusion, interference or conflict inhibition and response inhibition have the opposite effect on memory encoding. The memory for the target stimulus will be promoted when the target and distractor are presented in interference or conflict condition, whereas will be impaired when need to inhibit a response.

## 4 The neural mechanism for cognitive control affects memory encoding

### 4.1 the neural mechanism during task switching

According to previous studies, left inferior prefrontal cortex (LIPC) activation reflects successful encoding with more activating for subsequently remembered than forgotten items (Clark and Wagner, 2003; Wagner et al., 1998). The related research indicated that LIPC activation was stronger in the task-switching condition compared to single-task condition, while recognition of target items was worse in task-switching condition (Reynolds et al., 2004). Therefore, this suggests that LIPC activation negatively impacts item encoding when it is redirected to resolve task competitions.

ERP studies have demonstrated that brain activity in the encoding stage can predict memory performance, as reflected by the subsequent memory effect (SME) and the re-stimulus subsequent memory effect (Pre-SME). For SME, it is characterized by remembered items eliciting greater neural activation or evoking an enhanced late positive component (LPC) compared to forgotten items (Chakravarty et al., 2020; Collier et al., 2014; Dudukovic and Kuhl, 2017; Kim, 2011). Researchers have observed that items subsequently remembered elicited a more positive amplitude than forgotten items within the 300-600 ms following item presentation (Friedman and Johnson, 2000; Rugg, 1995; Wilding and Ranganath, 2012; Evans and Herron, 2019). In the task-switching paradigm, Richter and Yeung, 2016 found that items with high memory selectivity were associated with increased enhanced frontal negativity compared to items with low memory selectivity. Furtherly, the SME effect is affected by the type of memory, such as recognition and recall. Recognition relies on familiarity, whereas recall necessitates more detailed memory retrieval. The SME effect is more pronounced for recall than for familiarity, at least in young adults (Cansino and Trejo-Morales, 2008; Duarte et al., 2004; Friedman and Johnson, 2000; Mangels et al., 2001).

Regarding pre-SME, researchers often presented participants with a cue prior to the item and segmented the time window based on subsequent memory performance (e.g., remember or forget). Initially, Otten et al. (2006), (2010) used cue-task switching paradigm and the ERP results indicated that items subsequently remembered evoked enhanced frontal negativity compared to those subsequently forgotten. This frontal negativity is thought to reflect the involvement of LIPC in memory encoding and is related to subsequent memory recognition (Wagner et al., 1998). Koen et al. (2018) also employed a cue-task switch paradigm and discovered that younger participants exhibited a significant pre-SME at 1-2 s following cue presentation when the CSI was short. The pre-SME was regarded as related to preparation processes that promotes the formation of memory representations (Otten et al., 2006, 2010; Padovani et al., 2011). However, they did not concern the pre-SME for switch and repeat trials. While Padovani et al. (2013) have shown the pre-SME for switch trials appears later than repeat trials, possibly because switch trials need more time to finish effective task reconfiguration according to the current task requirements.

### 4.2 The neural mechanism during interference

fMRI studies have shown that memory encoding enhancement under interference or conflict conditions can be predicted by stronger activation in the dorsolateral prefrontal and parietal cortex (Eichenbaum et al., 2007; Krebs et al., 2015). Krebs et al. (2015) observed that incongruent trials, in contrast to congruent and neutral trials, were associated with significant activation in the dorsolateral prefrontal cortex (dlPFC). Additionally, stimuli that were subsequently remembered exhibited stronger activation in the dlPFC and the precuneus compared to those that were subsequently forgotten. The findings further suggested that the functional coupling between dlPFC and medial-temporal labe was enhanced for remembered vs. forgotten target stimuli in incongruent trials, reflecting that conflict-triggered prefrontal activation facilitates memory formation. Conflict, in particular, initiates top-down control mechanisms to mitigate interference by enhancing attention to the target stimulus. This process facilitates the encoding of the target stimulus, ultimately resulting in a more robust long-term representation.

Existing ERP researches on interference or conflict control and memory encoding mainly focus on the retrieval phase. During the memory recollection phase, following stimulus presentation, stimuli presented in incongruent trials elicited reduced frontal negativity (FN400) compared to congruent trials. This reduction is linked to familiarity-driven recognition processes (Curran and Hancock, 2007; Dulas and Duarte, 2013; Liu et al., 2016; Wang et al., 2021; Wolk et al., 2009). In contrast, the late posterior negativity (LPN), observed between 500-800 ms was associated with the recall of specific information (Curran and Cleary, 2003). Both Liu et al. (2016) and Wang et al. (2021) reported smaller frontal negativity and LPN in response to incongruent trials compared to congruent trials in an emotional conflict task. This suggests that the context in which stimuli are encoded influences familiarity processing during retrieval phases and the evaluation of retrieval outcomes.

### 4.3 The neural mechanism during response inhibition

Previous research has indicated that activity in the ventrolateral prefrontal cortex (VLPFC) can serve as predictor for impairments in memory encoding caused by response inhibition (Chiu and Egner, 2015b; Levy and Wagner, 2011). Consequently, VLPFC activation exhibits negative correlation with subsequent memory performance, particularly when selection processes redirect attention away from items intended for encoding (Otten and Rugg, 2002; Reynolds et al., 2004). Chiu and Egner, 2015b further revealed a negative correlation between the demand for inhibitory resources and the activation of the right VLPFC associated with successful encoding. Specifically, no-go stimuli that necessitate higher inhibitory resource demands were associated with diminished activation in the VLPFC. This suggest that response inhibition and stimulus encoding are in competition for limited attentional resources, with a greater allocation to response inhibition resulting in fewer resources available for stimulus encoding. Also, there must be other distinct mechanisms needs to be further studied.

## 5 Conclusion and future directions

There is a strong correlation between cognitive control and memory encoding, with particular emphasis on the impact of cognitive flexibility, inhibition control on memory encoding. Specifically, task switching can impair encoding for the target stimuli but promote encoding for non-target stimuli. Additionally, interference inhibition improves the encoding of stimuli with semantic conflict, while response inhibition impairs memory encoding. However, research on the intersection of cognitive control and memory is still in its developmental stage. In light of this, we attempt to provide an outlook for further exploration in this area.

First, it is imperative to integrate and refine the theoretical model. The common-resource account, interaction model, and stage-specific encoding theory offer distinct interpretations of the impact of cognitive control on memory encoding. Whereas, the existing theoretical model exhibits certain deficiencies, such as the applicability of the stage-specific encoding theory to the amalgamation of task switching and response inhibition, as well as the potential interactive effect of these two distinct cognitive controls on subsequent memory. It is remarkable to mention a persuasive framework-integrative framework, proposed by Muhmenthaler et al. (2023). They tend to believe that conflicts can be distinguished on attentional mechanisms and knowledge structures. According to this framework, conflicts based on attentional mechanisms may account for the inconsistent findings observed in Stroop, Flanker, and task switching paradigms. However, further empirical study is required to substantiate this framework.

Secondly, research has demonstrated that attention plays a crucial role in the processes of encoding and cognitive control. Consequently, it is imperative to investigate the influence of various factors that might affect attention mechanism on this process, such as emotional information. Existing studies primarily employed neutral stimuli to examine the interaction between cognitive control and memory encoding. However, several studies have reported different results when employing emotional stimuli. Dubravac et al. (2024) investigated the effects of task switching and emotional stimuli on selective attention and subsequent memory. Their findings indicated that negative distractors lowered memory selectivity, whereas negative targets increased memory selectivity. Nonetheless, they failed to observe an interaction between task switching and emotionality. Conversely, related research identified an interaction between Simon congruency and emotional valence. Specifically, participants were able to retrieve more positive words compared to negative words in the congruent condition (where the location of stimulus presentation is on the same side as the response hand), but this effect was diminished in the incongruent condition (Liu et al., 2016). Further experiments can be carried out to clarify the impact of conflict control processes on emotional memory.

Thirdly, researchers have focused on interesting findings, particularly in the realm of behavioral studies, which have often lacked a thorough exploration of the underlying processing mechanisms. Unfortunately, there have been few studies investing the mechanisms of cognitive control and memory encoding. For instance, Richter and Yeung (2016) using event-related potentials technology found that items subsequently remembered elicited enhanced frontal negativity compared to those subsequently forgotten. So far, there are limited studies focusing on the differences between remembered and forgotten items during early processing stages, such as attention and perceptual processing. Future research should employ technologies with higher temporal resolution or spatial resolution to clarify the underlying mechanisms of the interactions between cognitive control and memory encoding.
